# Dilated optic nerve sheath by ultrasound predicts mortality among patients with acute intracerebral hemorrhage

**DOI:** 10.1055/s-0043-1775885

**Published:** 2023-11-08

**Authors:** Francisco Antunes Dias, Maria Clara Zanon Zotin, Frederico Fernandes Alessio-Alves, Rui Kleber do Vale Martins Filho, Clara Monteiro Antunes Barreira, Otavio Costa Vincenzi, Paula Muñoz Venturelli, Gregoire Boulouis, Joshua Norkin Goldstein, Octavio Marques Pontes-Neto

**Affiliations:** 1 Universidade de São Paulo, Faculdade de Medicina de Ribeirão Preto, Hospital das Clínicas, Departamento de Neurociências e Ciências do Comportamento, Ribeirão Preto SP, Brazil.; 2 Universidade de São Paulo, Faculdade de Medicina de Ribeirão Preto, Hospital das Clínicas, Departamento de Medicina Interna, Divisão de Radiologia, Ribeirão Preto SP, Brazil.; 3 Harvard Medical School, Massachusetts General Hospital, Boston, MA, United States.; 4 Universidad del Desarrollo, Facultad de Medicina Clínica Alemana, Instituto de Ciencias e Innovación en Medicina, Centro de Estudios Clínicos, Santiago, Chile.

**Keywords:** Cerebrovascular Disorders, Stroke, Mortality, Intracranial Hypertension, Cerebral Intraventricular Hemorrhage, Optic Nerve, Ultrasonography, Transtornos Cerebrovasculares, Acidente Vascular Cerebral, Mortalidade, Hipertensão Intracraniana, Hemorragia Cerebral Intraventricular, Nervo Óptico, Ultrassom

## Abstract

**Background** Intracerebral hemorrhage (ICH) is a deadly disease and increased intracranial pressure (ICP) is associated with worse outcomes in this context.

**Objective** We evaluated whether dilated optic nerve sheath diameter (ONSD) depicted by optic nerve ultrasound (ONUS) at hospital admission has prognostic value as a predictor of mortality at 90 days.

**Methods** Prospective multicenter study of acute supratentorial primary ICH patients consecutively recruited from two tertiary stroke centers. Optic nerve ultrasound and cranial computed tomography (CT) scans were performed at hospital admission and blindly reviewed. The primary outcome was mortality at 90-days. Multivariate logistic regression, ROC curve, and C-statistics were used to identify independent predictors of mortality.

**Results** Between July 2014 and July 2016, 57 patients were evaluated. Among those, 13 were excluded and 44 were recruited into the trial. Their mean age was 62.3 ± 13.1 years and 12 (27.3%) were female. On univariate analysis, ICH volume on cranial CT scan, ICH ipsilateral ONSD, Glasgow coma scale, National Institute of Health Stroke Scale (NIHSS) and glucose on admission, and also diabetes mellitus and current nonsmoking were predictors of mortality. After multivariate analysis, ipsilateral ONSD (odds ratio [OR]: 6.24; 95% confidence interval [CI]: 1.18–33.01; *p* = 0.03) was an independent predictor of mortality, even after adjustment for other relevant prognostic factors. The best ipsilateral ONSD cutoff was 5.6mm (sensitivity 72% and specificity 83%) with an AUC of 0.71 ( *p* = 0.02) for predicting mortality at 90 days.

**Conclusion** Optic nerve ultrasound is a noninvasive, bedside, low-cost technique that can be used to identify increased ICP in acute supratentorial primary ICH patients. Among these patients, dilated ONSD is an independent predictor of mortality at 90 days.

## Resumo

**Antecedentes** A hemorragia intraparenquimatosa (HIP) aguda apresenta elevada morbimortalidade e a presença de hipertensão intracraniana (HIC) confere um pior prognóstico.

**Objetivo** Avaliamos se a dilatação do diâmetro da bainha do nervo óptico (DBNO) através do ultrassom do nervo óptico (USNO) na admissão hospitalar seria preditora de mortalidade.

**Métodos** Estudo multicêntrico e prospectivo de pacientes consecutivos com HIP supratentorial primária aguda admitidos em dois centros terciários. Ultrassom do nervo óptico e tomografia computadorizada (TC) de crânio foram realizados na admissão e revisados de forma cega. O desfecho primário do estudo foi a mortalidade em 3 meses. Análises de regressão logística, curva de característica de operação do receptor (ROC, na sigla em inglês) e estatística-C foram utilizadas para identificação dos preditores independentes de mortalidade.

**Resultados** Entre julho de 2014 e julho de 2016, 44 pacientes foram incluídos. A idade média foi 62,3 (±13,1) anos e 12 (27,3%) eram mulheres. Na análise univariada, o volume da HIP na TC de crânio, DBNO ipsilateral à HIP, glicemia, escala de coma de Glasgow (ECG) e NIHSS na admissão hospitalar, e também diabetes mellitus e não-tabagista foram preditores de mortalidade. Após análise multivariada, o DBNO ipsilateral à HIP permaneceu como preditor independente de mortalidade (odds ratio [OR]: 6,24; intervalo de confiança [IC] de 95%: 1,18–33,01; *p* = 0,03). O melhor ponto de corte do DBNO ipsilateral como preditor de mortalidade em 3 meses foi 5,6mm (sensibilidade 72% e especificidade 83%) e área sob a curva (AUC, na sigla em inglês) 0,71 ( *p* = 0,02).

**Conclusão** O USNO é um método não-invasivo, beira-leito, de baixo custo, que pode ser empregado para estimar a presença de HIC em pacientes com HIP supratentorial primária aguda. A presença de DBNO dilatada é um preditor independente de mortalidade em 3 meses nesses pacientes.

## INTRODUCTION

Acute intracerebral hemorrhage (ICH) is the most fatal and disabling form of stroke. 1 Numerous factors are known to be associated with worse outcomes, including admission ICH volume, older age, lower Glasgow coma scale (GCS) and presence of intraventricular hemorrhage (IVH). 2 However, none of these factors are modifiable. Increased intracranial pressure (ICP), on the other hand, has both been linked to worse outcomes, and is potentially treatable, making it a potential target for intervention. 3

Invasive ICP monitoring – that is, ventriculostomy or intraparenchymal microtransducers – is not routinely performed in ICH patients, since there is still no evidence of better clinical outcomes with this practice. 4 This procedure also imposes some risks such as infection, hemorrhage, and additional brain parenchyma injury. Thus, noninvasive methods for ICP estimation would be a very appropriate tool in this context and would allow for either invasive ICP monitoring/management to be targeted for those with known or suspected ICP elevation, and potentially help on the decision about indication of surgery and other invasive procedures. In this context, dilation of optic nerve sheath diameter (ONSD) diagnosed by transorbital optic nerve ultrasonography (ONUS) is an increasingly recognized marker of increased ICP in neurocritical patients. 5 6 We sought to evaluate whether dilated ONSD at hospital admission could be related to mortality among ICH patients.

## METHODS

This is a prospective, observational study with blinded longitudinal assessment of functional clinical outcomes. Consecutive patients with acute supratentorial primary ICH admitted to two tertiary stroke centers located in Brazil and Chile were prospectively recruited in this cohort. We planned to include all ICH patients admitted within 2 years and a convenience sample was set for 40 participants. The inclusion criteria were:

- ≥ 18 years old;
- supratentorial ICH diagnosed through cranial noncontrast computed tomography (NCCT) scan; and
- GCS > 5 on hospital admission.

The exclusion criteria were as follows:

- time of stroke onset of > 24 hours (when the exact time of stroke onset was not known, the last seen well time was used instead);
- immediate surgical intervention indicated by the neurosurgeon, including craniotomy and hematoma evacuation, ventriculostomy for hydrocephalus, or any other;
- secondary ICH (patients on regular anticoagulants and/or antiplatelets prescriptions were allowed to entry in this trial, but patients with hemorrhagic transformation of ischemic stroke, including thrombolytics, were excluded); and
- previous optic nerve pathology precluding accurate ONSD measurements.

**Figure 1:**
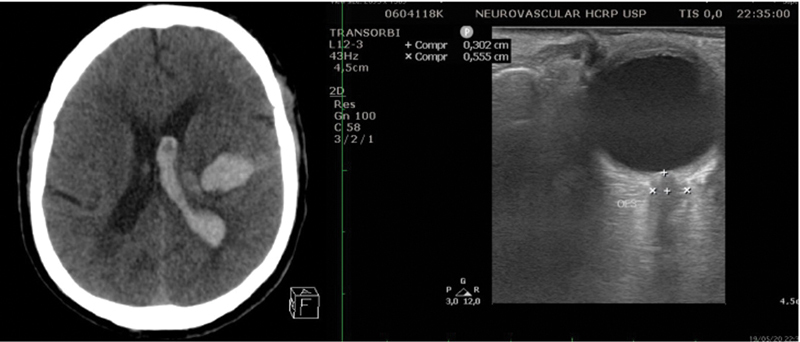
Baseline bedside ONUS on patient admission. (A) Cranial NCCT scan depicting a left ICH with volume of 11 ml and extensive left lateral ventricular hemorrhage. (B) ONUS showing ipsilateral dilated ONSD. Abbreviations: ICH, intracerebral hemorrhage; NCCT, non-contrast computed tomography; ONSD, optic nerve sheath diameter; ONUS, optic nerve ultrasonography.

Cranial NCCT scans and ONUS were both performed at hospital admission, with a time interval between exams of < 1 hour in all patients. Intracerebral hemorrhage volumes on cranial NCCT scans were retrospectively graded through consensus by one neuroradiologist (MCZZ) and two stroke neurologists (FAD, OCV), all of them with great expertise in acute cerebrovascular diseases assessment. Intracerebral hemorrhage volumes were quantified by the ABC/2 method, as previous described, 7 and NCCT scans were analyzed only after inclusion of all patients. Neuroimaging raters were blind to the clinical data. The ultrasound operator was also blinded to cranial NCCT scans findings but not for the clinical symptoms presentation of the patients. Optic nerve ultrasonography was performed with a 7.5-MHz linear array transducer by stroke neurologists on routine service, and patients were lying on the supine position. The ultrasound system was always set prior to patient examination to reduce the power output (according to the ALARA principle, that is, “as low as reasonably achievable”) and reduce possible damages to the ocular structures of the patient, as recommended by the World Federation for Ultrasound in Medicine and Biology (WFUMB). The ONUS protocol consists of 3 ONSD measurements at a depth of 3 mm behind the retina in each eye, as previously described ( Figure 1 ). 8 9 Optic nerve ultrasonography ipsilateral and contralateral to the ICH hemisphere (3 measurements each) and also the mean of both eyes (bilateral mean, total of 6 measurements) were separately obtained and results were blindly analyzed.

The primary outcome was 90-day all-cause mortality. For patients who survived the ICH acute phase and were discharged, a 90-day in-person visit at our outpatient clinic was appointed. If the patient could not present to the outpatient clinic, a telephone call was done. No patients were lost to follow-up. We investigated if ipsilateral, contralateral, and/or mean bilateral ONSD would be independent predictors of mortality. All baseline variables were obtained prospectively through patients or family members interview at hospital admission. The results were presented as means, medians, standard deviation, confidence intervals (quantitative variables), and frequencies and percentages (categorical variables). The normality of the variables was assessed using the Kolmogorov-Smirnov test. Student's t test or the nonparametric Wilcoxon-Mann-Whitney test was used to compare two groups in terms of quantitative variables. To compare the categorical variables of the groups, the chi-square test or Fisher's exact test was used. We used multivariate logistic regression ( *backward* ), receiver operating characteristic (ROC) curves and C-Statistics to identify independent predictors of mortality and best mean ONSD cutoffs. All variables on univariate analysis with a p-value < 0.05 were included in the final logistic regression models, excluding the collinear variables. We used the IBM SPSS Statistics for Windows, version 20 (IBM Corp., Armonk, NY, USA), and a p-value < 0.05 (two-sided) as the threshold for statistical significance.

## RESULTS

Between July 2014 and July 2016, 57 ICH patients were screened for the trial; 13 met the exclusion criteria, and the remaining were enrolled. Hence, 44 (27.3% female) were included on analysis. Their mean age was 62.3 (±13.1) years old, the median National Institute of Health Stroke Scale (NIHSS) was 17 (interquartile range [IQR]:11–21), the mean ICH volume was 29.6 ml (standard deviation [SD]: ± 34.6 ml), and the median time-to-arrival was 4 hours (IQR: 2.3–5.5). Overall mortality at 90-days was 41%.

**Figure 2:**
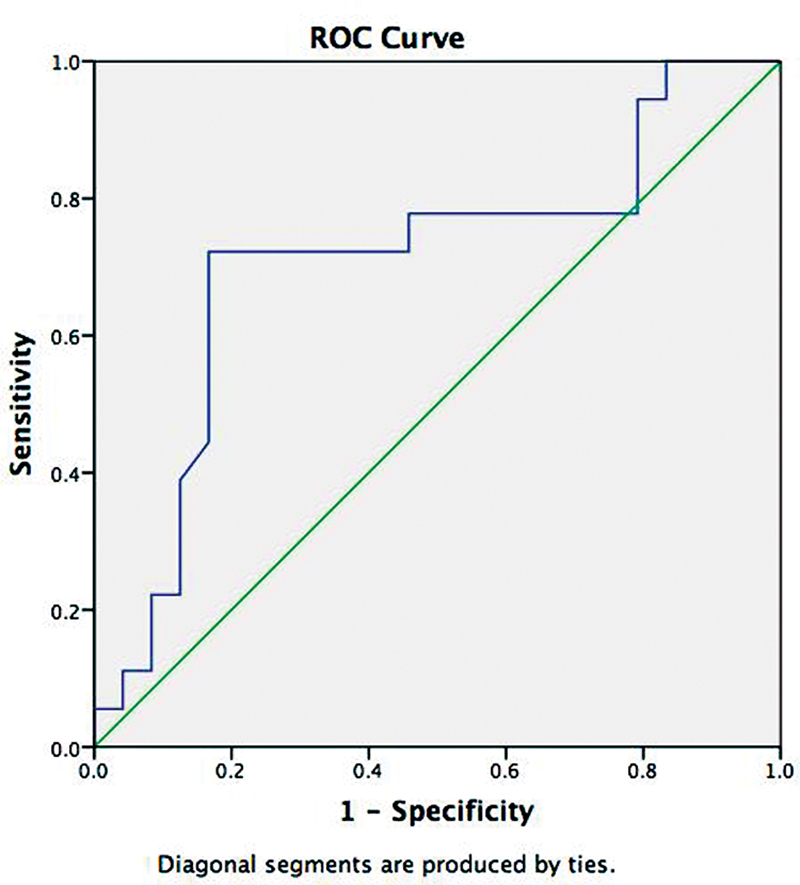
Abbreviations: AUC, area under the curve; ONSD, optic nerve sheath diameter; ROC, receiver operating characteristic. AUC (0.71; p=0.02) of ipsilateral ONSD measurement in ROC curve. Note: Diagonal segments are produced by ties.

None of the included patients received invasive ICP monitoring as standard management. On univariate analysis, ICH volume on admission NCCT scan, ipsilateral ONSD, GCS, NIHSS, admission blood glucose, diabetes mellitus, and current nonsmoking status were predictors of mortality ( Table 1 ). Conversely, contralateral ONSD ( *p* = 0.09) and mean bilateral ONSD ( *p* = 0.1) was not statistically associated with mortality ( Table 1 ). After multivariate analysis, ipsilateral ONSD (odds ratio [OR]: 6.24; 95% confidence interval [CI]: 1.18–33.01; *p* = 0.03) was an independent predictor of mortality, even after adjustment for admission ICH volume, age, GCS, and intraventricular hemorrhage ( Table 2 ). The ipsilateral ONSD had an area under the curve (AUC) of 0.71 ( *p* = 0.02) and the best ipsilateral ONSD cutoff was 5.6mm – with 72% sensitivity and 83% specificity – for predicting mortality at 3 months ( Figure 2 ).

**Table 1:** Baseline characteristics according to 90-day mortality ( *n* = 44)

		Alive ( *n* = 26)	Dead ( *n* = 18)	*p* - *value*
Female (%)		7 (27)	5 (28)	1.0
Age, years old (SD)		61 ± 13	64 ± 13	0.30
White (%)		18 (69)	14 (78)	0.75
Hypertension (%)		20 (77)	13 (72)	0.74
Diabetes (%)		2 (8)	7 (39)	0.02
Current smoker (%)		13 (50)	3 (17)	0.03
Current alcohol consumption (%)		16 (61)	9 (50)	0.54
Chronic liver disease (%)		0	2 (11)	0.16
Previous TIA/stroke (%)		0	3 (17)	0.06
Anticoagulation use (%)		1 (4)	3 (17)	0.29
Aspirin use (%)		2 (8)	3 (17)	0.39
Statin use (%)		1 (4)	3 (17)	0.29
Anti-hypertensive treatment (%)		12 (46)	10 (55)	0.76
Previous mRs 0–2 (%)		25 (96)	17 (94)	0.20
Time-to-arrival (h), median (IQR)		3.5 (2.7–4.4)	4 (2.2–6.3)	1.0
NIHSS, median (IQR)		15 (8–19)	19 (18–26)	0.003
GCS, median (IQR)		14 (11–15)	12 (11–14)	0.003
Systolic BP (mmHg), mean (SD)		182 ± 35	168 ± 48	0.35
Diastolic BP (mmHg), mean (SD)		109 ± 23	97 ± 24	0.23
Blood glucose (mg/dl), mean (SD)		129 ± 45	169 ± 62	0.01
ICH volume (ml)	mean (SD)	20 ± 25	44 ± 42	0.004
	median (IQR)	10 (5–23)	36 (10–59)	
Intraventricular hemorrhage (%)		13 (50)	11 (61)	0.55
Hematoma expansion (%)		4 (15)	5 (28)	0.45
ICH score (%)	0	10 (38)	2 (11)	0.24
	1	7 (27)	5 (28)	
	2	7 (27)	8 (44)	
	3	2 (8)	2 (11)	
	4	0	1 (6)	
ONSD (mm), median (IQR)	Ipsilateral	5.40 (5.30–5.56)	5.73 (5.41–6.12)	0.02
	Contralateral	5.26 (5.01–5.61)	5.40 (5.23–5.69)	0.09

Abbreviations: BP, blood pressure; GCS, Glasgow coma scale; ICH, intracerebral hemorrhage; IQR interquartile range; mRs, modified Rankin scale; NIHSS, National Institute of Health Stroke Scale; ONSD, optic nerve sheath diameter; SD, standard deviation; TIA, transient ischemic attack.

**Table 2:** Multivariate analysis of predictors of mortality

	OR	95% CI	*p* - *value*
**Ipsilateral ONSD**	6.24	1.18–33.01	0.03
**ICH volume on admission NCCT**	4.04	0.96–17.01	0.06
**Glasgow coma scale**	0.88	0.64–1.20	0.41

Abbreviations: CI, confidence interval; OR, odds ratio; ICH, intracerebral hemorrhage; NCCT, non-contrast computed tomography; ONSD, optic nerve sheath diameter.

## DISCUSSION

In the present prospective series of acute supratentorial primary ICH patients, ipsilateral ONSD measured by transorbital ONUS was an independent predictor of mortality at 90-days. Optic nerve ultrasonography is a relatively new technique for bedside, low-cost, reproducible, noninvasive ICP assessment. It has been recognized as an accurate tool to estimate increased ICP in a vast list of medical conditions. 5 6 However, to the best of our knowledge, this is the first study to investigate the role of ONSD measurement as a predictor of mortality in the setting of a pure acute supratentorial ICH cohort. Considering that invasive ICP monitoring is not a routine clinical practice for ICH patients, methods that allow non-invasive assessment of increased ICP have great potential for decision-making in clinical practice.

Our data support that ipsilateral ONSD was an independent predictor of mortality at 90 days, even after adjusting for important confounders. This could represent a real difference of ICP between cerebral hemispheres, that is, there could be a compartmentalization of ICP in acute ICH. Regarding contralateral ONSD, there was a tendency pointing toward significance ( *p* = 0.09; Table 1 ). Nevertheless, we cannot exclude that the lack of statistical significance was due to a small sample size. However, similar findings were also demonstrated in a study by Naldi et al., as asymmetry of ONSD between the two eyes was highly frequent in ICH patients. Among 20 patients with acute ICH included, asymmetry was identified in 14 (70%) cases, but both the highest unilateral ONSD value and the bilateral average values were considered accurate in identifying intracranial hypertension in their series, in contrast to our findings. 10

Recently, some studies of mixed stroke patients have shown good correlation between ONSD and clinical outcomes. Stroke patients with larger ONSD had higher mortality rates and poorer prognosis, in accordance with our results. 11 12 Hence, ONSD may be an accurate predictor of clinical outcomes in acute stroke patients. Optic nerve ultrasonography has also been applied with reliable accuracy in other clinical scenarios to predict in-hospital mortality, such as acute severe COVID-19 patients admitted to intensive care units (ICUs). 13 In this very interesting study, critically ill patients who deceased had a significantly higher ONSD compared with survivors. The authors correlated these findings with a probable increase in ICP primarily through cerebral vasodilatation and increases in cerebral blood volumes caused by hypoxic hypercapnia resulting from COVID-19 pneumopathy. 13

Furthermore, ONSD was directly correlated to invasive ICP measurement by lumbar puncture in the setting of correction of intracranial hypertension with mannitol infusions in ambulatory patients. Optic nerve sheath diameter was also correlated with invasive ICP before and after resolution of the underlying cause of elevated ICP in acute neurocritical patients. Dynamic variations in ICP could be precisely depicted by ONUS in both studies. 14 15 Thus, we believe ONUS may be an adjuvant tool to dynamically and noninvasively assess ICP in ICH patients. However, ONUS does have limitations. The lack of a reliable ONSD cutoff value for detecting increased ICP accurately is still disappointing, with reports ranging from 4.8mm to 6.3mm. 6 This variability may be related to heterogeneity regarding definition of intracranial hypertension (ICP > 20mmHg, ICP > 20cmH _2_ O, or ICP > 25cmH _2_ O) in previous studies. 6 It could also be explained by the fact that ONUS demands an experienced operator, due to some common pitfalls and artifacts. Nevertheless, the learning curve for adequately performing an ONUS exam is relatively short and ONUS may be a useful tool to detect increased ICP, particularly in a nonquantitative binary mode. 5

In addition to measuring ONSD by ONUS, other noninvasive ultrasonographic methods have also been studied for identifying intracranial hypertension. Among these, the evaluation of pulsatility index (PI) and noninvasive estimation of intracranial pressure (e-ICP), both derived from flow velocities of major intracranial arteries (particularly the middle cerebral arteries), as well as the analysis of PI and velocity of venous flow of the superior sagittal sinus or straight sinus, have shown encouraging results. 16 17 18 However, in a study by Robba et al., which compared all these ultrasonographic methods for identifying intracranial hypertension, ONSD measured by ONUS showed the most significant correlation with invasive measurements of ICP. 19 In another study by Robba et al, e-ICP showed the best correlation with invasive ICP instead. 20 In both studies, there was an additional gain in accuracy when two ultrasonographic methods were analyzed together, especially the combination of ONSD and e-ICP, with an AUC = 0.91 (95%CI: 0.84–0.97). 19 20 Thus, multimodal monitoring, including more than one ultrasonographic method, along with neuroimaging, is likely the most appropriate approach for the correct noninvasive identification of intracranial hypertension in neurocritical patients. In addition to sonography, other noninvasive methods have been developed to identify intracranial hypertension. 16 Probably, the most promising one is a device which quantifies minimal cranial bone deformations using specific sensors, generating ICP waveforms noninvasively. A parameter derived from noninvasive ICP waveforms could predict mortality and had good correlation with intracranial hypertension diagnosed through invasive ICP in acute traumatic brain injury patients. 21

The present study has several limitations. We excluded ICH patients admitted with GCS 3–5, as these patients have been consistently linked to bad prognosis. Furthermore, the mean ICH volume of our cohort was 29.6 ml. Thus, our results should not be extrapolated to high-volume, severely impaired ICH patients. In this cohort, ipsilateral ONSD ≥ 5.6mm was the best cutoff value for predicting mortality. This finding may reflect possible significant differences in ICP levels between affected and nonaffected cerebral hemispheres in ICH patients, as discussed before. However, in our protocol, the ONUS operator was not blinded to clinical presentation. Thus, we cannot exclude a measurement bias. Nonetheless, our strict and objective protocol of ONSD measurements significantly lowered this limitation. Moreover, despite the valuable collaboration other expert neurosonologists, the first author (FAD) was responsible for conducting over 90% of all ONUS examinations, due to limitations of resources and available research personnel. Therefore, no interrater analysis could be performed with the recruited patients. Invasive ICP monitoring is not mandatory in both ICH institutional protocols, so the present study was not powered to analyze the best ONSD cutoff to identify increased ICP. Our series has also a relatively small sample size from only two centers, and our findings must be confirmed in larger multicenter ICH cohorts. Future studies should investigate if significant ICP differences do exist between cerebral hemispheres and if invasive ICP monitoring is clinically useful in ICH patients with dilated ONSD suggesting increased ICP. However, our results suggest that ONUS is a promising technique for routine clinical practice, and it should be further explored in the management of ICH patients.

In conclusion, ONUS is a noninvasive, bedside, low-cost technique that may be used to estimate the presence of increased ICP in patients with acute supratentorial primary ICH. Among these patients, ipsilateral dilated ONSD is an independent predictor of mortality at 90-days.
